# Self-care related to the performance of occupational roles in patients under antineoplastic chemotherapy treatment*

**DOI:** 10.1590/1518-8345.4092.3421

**Published:** 2021-05-21

**Authors:** Leidiane Mota De Oliveira Chagas, Fabiano Henrique Oliveira Sabino, Maria Helena Barbosa, Heloisa Cristina Figueiredo Frizzo, Luana Foroni Andrade, Elizabeth Barichello

**Affiliations:** 1 Universidade Federal do Tringulo Mineiro, Instituto de Cincias da Sade, Uberaba, MG, Brazil.; 3 Universidade Federal de Sergipe, Lagarto, SE, Brazil.

**Keywords:** Role Playing, Self Care, Drug Therapy, Neoplasms, Integrality in Health, Social Support, Desempenho de Papis, Autocuidado, Tratamento Farmacolgico, Neoplasias, Integralidade em Sade, Apoio Social, Desempeo de Papel, Autocuidado, Quimioterapia, Neoplasias, Integralidad en Salud, Apoyo Social

## Abstract

**Objective:**

to analyze and correlate occupational roles, symptoms and self-care capacity in oncologic patients seen at the chemotherapy service of a university hospital.

**Method:**

cross-sectional study, in which the instruments were applied sociodemographic and clinical questionnaire, M.D Andersons Symptom Inventory - core, Appraisal of Self Care Agency Scale-Revised and Occupational Paper Identification List to oncologic patients seen in the chemotherapy service of a university hospital. Data analysis included absolute and relative frequency tables and multiple linear regression, adopting a significance level of =0.05.

**Results:**

the sample showed capacity for self-care operationalized with an average of X=57.8. In the correlation between the degree of importance of the occupational papers and the scores of the evaluation instrument for self-care was found statistical significance in the papers of volunteer (r=0.26; p=0.02) and friend (r=0.33; p= <0.001). The linear regression showed that the greater the interference of symptoms in life activities (=0.20; p=0.05) and greater the importance of the role of friend (p=0.001; p=0.43), the higher the rates of self-care.

**Conclusion:**

the operationalization of self-care can be directly related to the degree of importance attributed to the performance of social roles.

## Abstract (pt)

**Objetivo:**

analisar e correlacionar os papis ocupacionais, os sintomas e a capacidade de autocuidado em pacientes oncolgicos atendidos no servio de quimioterapia de um hospital universitrio.

**Mtodo:**

estudo transversal, no qual foram aplicados os instrumentos questionrio sociodemogrfico e clnico, Inventrio de Sintomas do M.D Anderson - core, escala de avaliao da capacidade do autocuidado *Appraisal of Self Care Agency Scale-Revised* e Lista de Identificao de Papis Ocupacionais em pacientes oncolgicos atendidos no servio de quimioterapia de um hospital universitrio. A anlise dos dados incluiu tabelas de frequncias absolutas e relativas e regresso linear mltipla, adotando nvel de significncia de =0,05.

**Resultados:**

a amostra apresentou capacidade para o autocuidado operacionalizada com mdia de X=57,8. Na correlao entre o grau de importncia dos papis ocupacionais e os escores do instrumento de avaliao para o autocuidado foi encontrada significncia estatstica nos papis de voluntrio (r=0,26; p=0,02) e de amigo (r=0,33; p= <0,001). A regresso linear mostrou que quanto maior a interferncia dos sintomas nas atividades de vida (=0,20; p=0,05) e maior a importncia do papel do amigo (p=0,001; p=0,43), maiores os ndices de autocuidado.

**Concluso:**

a operacionalizao do autocuidado pode estar diretamente relacionada ao grau de importncia atribudo ao desempenho de papis sociais.

## Abstract (es)

**Objetivo:**

analizar y correlacionar los roles ocupacionales, los sntomas y la capacidad de autocuidado en pacientes oncolgicos atendidos en el servicio de quimioterapia de un hospital universitario.

**Mtodo:**

estudio transversal, en el que se aplicaron los instrumentos cuestionario sociodemogrfico y clnico, Inventario de Sntomas de M.D. Anderson - core, escala de evaluacin de la capacidad de autocuidado *Appraisal of Self Care Agency Scale-Revised* y Lista de Identificacin de Roles Ocupacionales en pacientes oncolgicos atendidos en el servicio de quimioterapia de un hospital universitario. El anlisis de los datos incluy tablas de frecuencias absolutas y relativas y regresin lineal mltiple, a un nivel de significancia de =0,05.

**Resultados:**

la muestra ha mostrado capacidad para el autocuidado operacionalizada, con un promedio de X=57,8. En la correlacin entre el grado de importancia de los roles ocupacionales y las puntuaciones del instrumento de evaluacin para el autocuidado, se encontr significancia estadstica para los roles de voluntario (r=0,26; p=0,02) y de amigo (r=0,33; p= <0,001). La regresin lineal mostr que cuanto mayor es la interferencia de los sntomas en las actividades de la vida (=0,20; p=0,05) y cuanto mayor la importancia del rol del amigo (p=0,001; p=0,43), ms altos son los ndices de autocuidado.

**Conclusin:**

la operacionalizacin del autocuidado puede estar directamente relacionada con el grado de importancia atribuido al desempeo de los roles sociales.

## Introduction

The antineoplastic chemotherapy treatment can be long and disabling, as well as involving environmental and social aspects, which aggravate and alter the functional capacity and various activities of life^(^ 1 ^-^ 2 ^)^.

Adverse events related to cancer treatment are very frequent and 86% of patients report experiencing, at least, one side effect^(^ 3 ^)^. Even with technological advances, the treatment has caused adverse events such as myelodepression, alopecia, nausea, vomiting, diarrhea, dyspnea, pain, loss of appetite, cognitive changes, cachexia, depression and fatigue, interfering with adherence or making the patient give up the chemotherapy treatment^(^ 4 ^-^ 5 ^)^.

It becomes relevant to guide the oncologic patient about his/her health condition and treatment, promoting his autonomy and self-care, in search of the prevention of disabilities resulting from his chronic condition. The Orems Theory of Self-Care bases the interventions on self-care, not only among the nursing team but, also, serves as a theoretical subsidy for the occupational therapists. This concept presents itself as a complex, innate ability, performed throughout life, with a focus on recognizing and performing personal needs^(^ 6 ^-^ 7 ^)^. By providing self-confidence in performing self-care in cancer patients during chemotherapy, a positive impact on side effects, such as fatigue, can be achieved^(^ 8 ^)^.

The adverse events of cancer and chemotherapy imply changes in the performance of daily activities which, in turn, impact the performance of occupational roles. One of the main theoretical references of occupational therapy, the Human Occupation Model (HOM), describes occupational actions, such as the identity and expectation of the person in relation to his/her attributions in society. Occupational roles include being a student, worker, caregiver, performing leisure, domestic and family activities, participating in groups of friends, religious or other^(^ 9 ^)^.

Studies covering self-care and the performance of occupational roles in chemotherapy patients contribute to evidence-based practice, since national and international research aimed at analyzing the consequences of chemotherapy treatment on the capacity of self-care and the performance of occupational roles is scarce. Furthermore, the public health policies available for the person with cancer emphasize the need to expand care, instituting multidisciplinary practices, in order to reduce the losses resulting from the disease and treatment, often responsible for costly treatments and burdens to the health system and society^(^ 9 ^-^ 11 ^)^.

Given the above, this article aims to analyze and correlate the occupational roles, symptoms and self-care capacity in oncologic patients seen at the chemotherapy service of a university hospital.

## Method

This is a quantitative study, with cross-sectional design, conducted at the chemotherapy center of a university hospital, located in a medium-sized city (Uberaba) in the in the region known as Tringulo Mineiro, in Minas Gerais (MG), Brazil, between the months of March and July 2017, with 100 patients being interviewed who were undergoing antineoplastic chemotherapy treatment.

The inclusion criteria considered in this research were: adults and elderly of both sexes, with preserved cognitive capacity and submitted to, at least, one cycle of antineoplastic chemotherapy and diagnosed with primary cancer, considering that metastasis has worse prognosis and treatment. Individuals with recent postoperative period (up to 40 days) were excluded.

**Figure 1:**
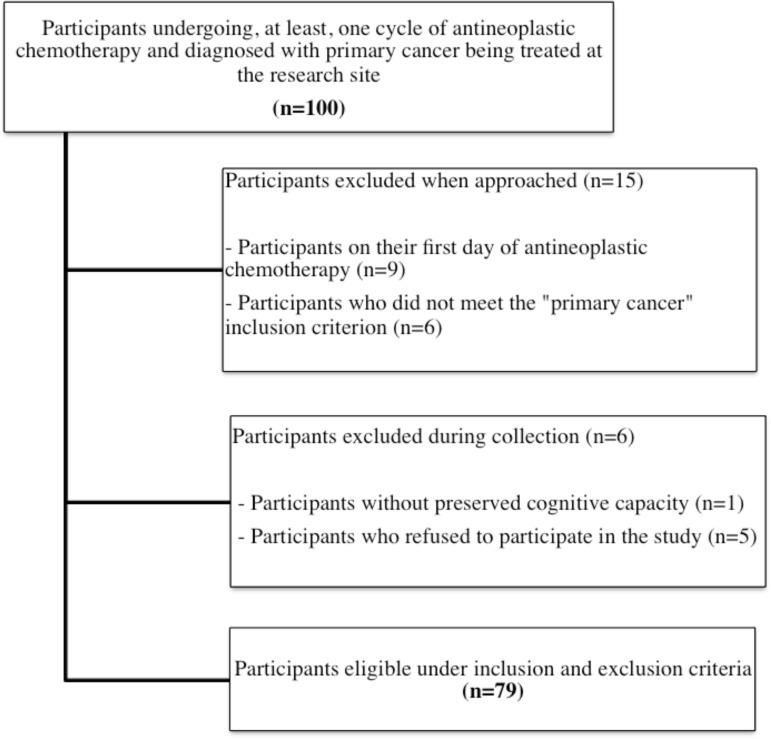
Selection of eligible participants for the study (n=79) from a population of individuals undergoing antineoplastic chemotherapy treatment. Uberaba, MG, Brazil, 2017

Figure 1 shows the flowchart of participants during the collection period.

An instrument was developed by the researchers themselves to address socio-demographic and clinical issues, with the following information: age, sex, marital status, years of study, family income, occupation, cohabitation and religious habits. The time and type of chemotherapy treatment, adverse events of chemotherapy, location of cancer, comorbidity or other diseases and the use of psychoactive substances were used to define the type of treatment and clinical conditions of health.

The M.D Andersons Symptom Inventory - core (MDASI) was used to evaluate the presence of individual or multiple symptoms related to cancer and its treatment. Translated and validated for the Brazilian population, the instrument presented adequate psychometric properties, with General Cronbach MDASI alpha of 0.85, symptoms of 0.78 and interferences of 0.79. The factorial analysis of 0.79 evidenced adequacy of data^(^ 10 ^-^ 12 ^)^.

As for the structure, part I of MDASI is composed of 13 questions that investigate the presence and intensity of symptoms in the patients life, being zero without symptoms and 10 a symptom as strong as you can imagine. Part II addresses, in five questions, how much the symptoms described in part I affect life, which varies from zero to symptom did not interfere in the life of the patient to ten interfered completely. For not having a general score, the result is the average of the sum of the scores of the two parts of the instrument.

The Occupational Paper Identification List instrument was used to evaluate the performance of the participants life roles and the importance attributed to them, having been translated and validated into Brazilian Portuguese^(^ 11 ^)^. This instrument has been used in researches from the calculation of the frequency of the different occupational roles in the past, present and future times, as well as the degree of importance.

The participant points out among the ten roles listed (student, worker, volunteer, caregiver, domestic service, friend, family member, religious, hobby/amateur and participant in organizations) about their performance in the past, present and future, giving the degree of importance for each of the roles, considering the score 1 for none, 2 for some and 3 for much importance.

Subsequently, the roles are listed in eight categories: role played only in the past, loss of role only in the present, role played only in the present, role played until the present, role played to date, new role in the future, continuous role and role absent from analysis. This is done to establish standards of performance and the degree of importance of each role for the participant.

The distribution and frequency of the instrument data are automatically generated by a database created by the person responsible for the validation of the scale in Brazil.

This research covered the roles played in the past and which have changed in the present due to the occurrence of the chemotherapy event, considering also the degree of importance attributed to each role. Next, the results were correlated to the results of the self-care scale.

The Self-Care Capacity Assessment Scale (SCCAS) was translated, adapted and validated for Brazil with Cronbach alpha of 0.74, intra-class correlation coefficient in the test and retest of 0.81 and inter-observer analysis of 0.84 in its psychometric properties. The scale has 15 items and the answers vary in a Likert type scale. The total score goes from 15 to 75 points and the closer to 75, the more operationalized the capacity of self-care is^(^ 7 ^)^.

The study variables were submitted to statistical analysis and testing in the Statistical Package for the Social Sciences (SPSS) software, version 21.0. The analysis included absolute frequency tables. For the bivariate analysis, the t-test was performed for the independent dichotomous samples and the Spearman correlation for the ordinal variables. The simultaneous influence of demographic, clinical and role predictors on self-care, was performed through multiple linear regression analysis. The level of significance considered in this work was p0,05.

The research respected the ethical precepts provided by Resolution 466/2012 and had an approved opinion number 1,941,852.

## Results

A total of 79 patients participated in the study, 50.6% of whom were women, 54.4% were elderly, the age range was 23 to 86 years, with a standard deviation (SD) of 13.5 and a mean age of 56.9 years. Regarding schooling, 45.6% had up to 4 years of study and 8.9% of these were illiterate. Regarding occupation, 58.2% were retired. The prevailing cancer was that of the digestive system (35.4%), followed by breast, uterus and prostate cancers (20.3%).

Fatigue and lack of appetite were reported by 64.6%, followed by nausea with 58.2%. The highest averages obtained were for symptoms of concern (3.82) and fatigue (3.72). The area of life activities in general, showed greater interference in the participants, due to the appearance of symptoms, prevailing at 78.5% (1.22 0.41). The capacity for work (including domestic chores) suffered interference from the symptoms for 73.4% (1.270.44) of participants.

The analysis of the performance of occupational roles showed that the roles of worker (84%) and domestic service (86%) presented a greater loss of performance in the present, if compared to performance in the past.

When considering the period of occurrence of the chemotherapy event in the present, the role of family member was the most played (82%). As a result of the degree of importance of occupational roles, it was obtained that the role of volunteer was 42% of some importance and the role of family member was indicated as very important by 92%.

The frequency analysis of the total SCCAS scores showed capacity for operational self-care with an average of X=57.8 and SD=5.2 and the closer to 75, the greater the capacity for self-care (Table 1).

**Table 1:** Descriptive analysis of the evaluation for the self-care capacity of participants (n=79) in antineoplastic chemotherapy treatment. Uberaba, MG, Brazil, 2017

Selfcare score	%
Average	57.8
Median	58.0
Standard model	5.2
Minimum	43.0
Maximum	67.0

The result of the answers I fully agree was 70.9% on the items of the instrument that related to seeking to develop the best ways to care for oneself and on the ability to obtain information, when identified health threats.

To meet the objective of the research, correlations were made between the scores of the self-care dependent variable on the means of sociodemographic variables, the interference of symptoms in the activities of life of the person (MDASI-Part II) and the degree of importance of occupational papers (List of Identification of Occupational Papers).

The results of the comparison analysis from the t-test showed that, statistically, the relationship between the sociodemographic variables and the components of the symptom evaluation instrument, related to the self-care scores, was not significant. However, it was possible to find that the greater the interference of the symptoms in the life activities of the person with cancer in antineoplastic chemotherapy treatment, the greater its capacity for self-care (SD of 5.17; p=0.08).

In the correlation between the degree of importance of occupational roles and the scores of the evaluation instrument for self-care, statistical significance was found in the roles of volunteer (r=0.26; p= 0.02) and friend (r=0.33; p= <0.001). These results showed that the more important the performance of these roles for the participant, the greater is his self-care (Table 2).

**Table 2:** Spearman correlation between the self-care scores, according to the degree of importance of roles in the lives of the participants (n=79) in antineoplastic chemotherapy treatment. Uberaba, MG, Brazil, 2017

Roles	Self-care	
	*r*_s_ *	*p* ^[Table-fn TFN2]^
Student	0.05	0.66
Worker	0.03	0.79
Volunteer	0.26	0.02
Caregiver	0.17	0.14
Housekeeping	-0.04	0.72
Friend	0.33	<0.001
Family member	0.12	0.29
Religious leader	0.15	0.18
Hobby/amateur	0.02	0.89
Participant in organizations	0.17	0.13

* *r*_s_= Magnitude of Spearman's correlation coefficient;

p= Spearman's correlation at significance level

For linear regression, three variables were selected that had a higher frequency in this survey, being sex, interference of symptoms in general activities and the importance of the role of friend over the self-care variable (Table 3).

**Table 3:** Linear regression of the self-care score on sex predictors, interference of symptoms in general activities and importance of the role of participant's friend (n=79) in antineoplastic chemotherapy treatment. Uberaba, MG, Brazil, 2017

	*	*p* ^[Table-fn TFN4]^
Sex	-0,13	0,19
Interference of symptoms in general activities	0,20	0,05
Importance of the role of friend	0,43	<0,001

* = Beta Coefficient;

*p* = Logistic regression, level of significance

The result obtained showed that the greater the interference of symptoms in life activities (=0.20; p=0.05) and the greater the degree of importance attributed to the role of friend (=0.43; p0.001,), the greater the self-care of participants.

## Discussion

Studies with the adult and elderly population served in the Brazilian Unified Health System (UHS) and that perform antineoplastic chemotherapy treatment present sociodemographic data consistent with those of this research^(^ 13 ^-^ 14 ^)^. In the oncologic practice, sociodemographic variables, such as gender, age, income, schooling, cohabitation and occupation, must be considered, due to its influence in the early detection of cancer and the consequent search for the health service, facilitating the institution of the best treatment and even the survival to cancer. In turn, early detection is permeated by population health education, which should be focused on self-care practices, even before the diagnosis of an illness^(^ 15 ^-^ 16 ^)^.

Among the oncologic adult and elderly patients attended at UHS, researches point out that, when seeking health care, the disease is in a more advanced stage, if compared to the stage of those seeking health care in developed countries. For the health system and society, this factor may generate burdens due to increased disabilities and loss of functionality, leading to early retirement and decreased health-related quality of life (HRQL) and hindering chances of survival^(^ 1 ^,^ 17 ^)^.

By accepting these issues, it is the duty of health professionals to provide guidance and education on self-care practices during chemotherapy treatment in order to prevent the adverse effects of antineoplastic chemotherapy and to increase the chances of survival and higher HRQL^(^ 18 ^)^.

Self-care practices can be classified as passive, when the professional performs the care without the help of the patient; collaborative, when the professional and the patient perform together health care actions and active, related to the practices and actions performed by the person without the help of a health care professional^(^ 19 ^)^.

However, the persons involvement with cancer in self-care practices may vary according to their socioeconomic situation, the treatment performed, the socio-family support and their life perspectives. The health professional should encourage the significant adoption of habits and behaviors that favor the active practice of self-care throughout the life of the sick person and his/her family, engaging him/her in these actions beyond the treatment period^(^ 19 ^)^.

In order for healthy habits and behaviors to occur in people with cancer, it is important that these patients psychologically face the new lifestyle, adjusting to the new health condition and the challenges experienced as a result of the chronic disease, in addition to psychological adaptation, in order to become resilient to the stressful variables that impact on behavior and interaction with the environment^(^ 20 ^)^.

In this study, the concepts of psychological adjustment and adaptation can be linked to the good operationalization of self-care among the participants, as they reported looking for the best ways to care for themselves and the ability to obtain information when they feel health threats.

The greater the interference of symptoms in general life activities, the higher was the participants self-care score. Research on the engagement of people with cancer in self-care actions cites that the patients mobilization in the face of the stress caused by the disease and its treatment can be motivated by threats to independent performance in their main life activities. This process, known as self-regulation, concerns the active commitment to health care during the treatment process of the chronic disease, such as cancer^(^ 20 ^)^.

In addition, the literature brings how much social and family support is indispensable when it comes to favorable responses to treatment and life perspectives of the person with cancer. Such psychological support, offered by the social and family support network, helps in the management of feelings and emotions, which may limit or engage the person in face of the diagnosis and treatment^(^ 20 ^-^ 21 ^)^.

In helping to have a positive perspective to face the changes caused by cancer, studies report that the role of friend has different characteristics than that of family members, because he is less involved in the daily stress caused by the disease and its treatment. This makes him more able and emotionally prepared to offer support and support to patients undergoing treatment, engaging them in a more active posture through the necessary adaptations^(^ 20 ^,^ 22 ^)^.

Although several demographic variables were analyzed as predictors of the operationalization of self-care, no sociodemographic variable was statistically significant in this study. Nevertheless, the sex of the individual, socioeconomic condition and schooling are commonly found in the literature, as influencing self-care ability and should be considered in clinical practice^(^ 21 ^,^ 23 ^)^.

It is valid for health professionals to know the conditions and variables that may protect and/or influence the adaptation of the chronic patient to his new health condition^(^ 20 ^)^. It is also stated that, even after a diagnosis such as cancer, it is possible that many patients manage to resign their lives, adopting habits and behaviors in order to restructure themselves and live a more active and healthy life, by committing themselves, even more, to their occupations and their family and social relationships^(^ 24 ^)^.

The results of this study may suggest that the attention to the health of the person with cancer should cover, not only the management of symptoms, but also the various life contexts, in which the disease and the treatment itself can cause serious functional damage. Researches that substantiate actions in this sense meet the preconditions of public health policies aimed at the humanized treatment of the person with cancer, which point out the importance of an integrated care carried out by a multidisciplinary health team, so that rehabilitation increases the chances of survival to the cancer linked to HRQL.

Since this is a cross-sectional study, the data should not be generalized to other populations. In addition to the limitations of the study design, it is believed that the number of participants and the diversity of cancer types made it difficult to perform a statistical analysis of correlation between the variables. It is suggested that further clarification of the relationship between possible alterations in the performance of occupational roles and the capacity for self-care in people with cancer, even before the beginning of antineoplastic chemotherapy treatment, be investigated in future studies of a longitudinal methodological nature.

This study can contribute to the knowledge that the interference of symptoms resulting from antineoplastic chemotherapy treatment in life activities and the greater degree of importance attributed to the occupational roles of volunteer and friend are relevant predictors, to be related to the operationalization of self-care.

## Conclusion

The results showed that there are symptoms that presented greater interference in relation to the area of life when these involved the activities in general. When analyzing the performance of occupational roles, the role of worker and the role of domestic service showed a greater loss of performance in the present, compared to performance in the past. The result obtained in linear regression showed that the greater the interference of symptoms in life activities and the degree of importance attributed to the role of friend, the greater is the self-care of the participants. There are symptoms that can harm the patient in antineoplastic chemotherapy treatment and compromise the self-care and role playing.
